# The Functional and Antiviral Activity of Interferon Alpha-Inducible IFI6 Against Hepatitis B Virus Replication and Gene Expression

**DOI:** 10.3389/fimmu.2021.634937

**Published:** 2021-04-01

**Authors:** Muhammad Sajid, Hafiz Ullah, Kun Yan, Miao He, Jiangpeng Feng, Muhammad Adnan Shereen, Ruidong Hao, Qiaohong Li, Deyin Guo, Yu Chen, Li Zhou

**Affiliations:** ^1^ State Key Laboratory of Virology, Modern Virology Research Center, College of Life Sciences, Wuhan University, Wuhan, China; ^2^ Ministry of Education Key Laboratory of Tropical Disease Control, The Infection and Immunity Center (TIIC), School of Medicine, Sun Yat-sen University, Shenzhen, China; ^3^ Animal Biosafety Level III Laboratory at Center for Animal Experiment, Wuhan University, Wuhan, China

**Keywords:** HBV, interferon-stimulated genes, antiviral activity, CHIP, EMSA, IFI6

## Abstract

Hepatitis B virus is an enveloped DNA virus, that infects more than three hundred and sixty million people worldwide and leads to severe chronic liver diseases. Interferon-alpha inducible protein 6 (IFI6) is an IFN-stimulated gene (ISG) whose expression is highly regulated by the stimulation of type I IFN-alpha that restricts various kinds of virus infections by targeting different stages of the viral life cycle. This study aims to investigate the antiviral activity of IFI6 against HBV replication and gene expression. The IFI6 was highly induced by the stimulation of IFN-α in hepatoma cells. The overexpression of IFI6 inhibited while knockdown of IFI6 elevated replication and gene expression of HBV in HepG2 cells. Further study determined that IFI6 inhibited HBV replication by reducing EnhII/Cp of the HBV without affecting liver enriched transcription factors that have significant importance in regulating HBV enhancer activity. Furthermore, deletion mutation of EnhII/Cp and CHIP analysis revealed 100 bps (1715-1815 nt) putative sites involved in IFI6 mediated inhibition of HBV. Detailed analysis with EMSA demonstrated that 1715-1770 nt of EnhII/Cp was specifically involved in binding with IFI6 and restricted EnhII/Cp promoter activity. Moreover, IFI6 was localized mainly inside the nucleus to involve in the anti-HBV activity of IFI6. *In vivo* analysis based on the hydrodynamic injection of IFI6 expression plasmid along with HBV revealed significant inhibition of HBV DNA replication and gene expression. Overall, our results suggested a novel mechanism of IFI6 mediated HBV regulation that could develop potential therapeutics for efficient HBV infection treatment.

## Introduction

The infection of the Hepatitis B virus (HBV) has a significant community health concern for the past few decades (1–3). About three hundred and sixty million people are chronically infected with severe liver diseases due to HBV (4, 5). Current treatment for chronic HBV infection is restricted to pegylated type 1 IFN and nucleotides/nucleosides analogs (6). However, current treatment for chronic HBV could only suppress HBV replication but unable to cure the disease due to the presence of minichromosomal cccDNA (7).

HBV is a noncytopathic and hepatocytotropic enveloped DNA virus that belongs to *Hepadnaviridae* family (8). The genome of HBV contains a greatly condensed genetic structure comprised of 3.2-kb partially closed relaxed circular double-stranded DNA (rcDNA). Upon hepatocytic infection through high-affinity binding with sodium taurocholate cotransported polypeptide (NTCP) receptor, rcDNA is converted into long-lived minichromosomal cccDNA by different host cellular enzymes in the nucleus and acts as a template for the transcription of HBV RNAs that serves as the primary transcript for the regulation and synthesis of all viral transcripts (Pregenomic, Precore, preS1, preS2, and X RNA transcript) (9, 10). Seven different HBV proteins are translated from these transcripts: HBeAg (hepatitis B e antigen), HBc (core protein), HBV polymerase (pol), the small (S), medium (M), large (L) envelope glycoprotein, and HBx protein (11, 12). The transcriptional activity of HBV RNAs is regulated by four HBV specific promoters (core, preS1, preS2, and X), two enhancer elements (EnhI and EnhII), and a negative regulatory cis-acting element (13). The cytokines and host nuclear factors are key elements to regulate the transcriptional activities of HBV promoters including STAT1 (signal transducer and activator of transcription), RXR (retinoid X receptor), and HNF-4 (hepatocyte nuclear factor 4) (14).

The type I IFN (IFN-α, β, and ε) is considered as the potent antiviral cytokine in vertebrates and has been widely used in the treatment of chronic HBV. Upon viral infection, the type I IFN induction carried out through binding of its type I IFN receptor (IFNAR1 and IFNRA2), subsequently activation of the cascade of JAK/STAT signaling pathway, leading to the induction of about 300 IFN-stimulated genes in the host nucleus that acts as an antiviral function, inhibits different stages of viral replication, transcription and post-transcriptional events (15–17). Since IFN-α therapy has some side effects or limitations ranging from mild clinical symptoms to significant mortality and morbidity (18), it is urgent to develop IFN- α related therapy that has the anti-HBV effect without significant limitations and clinical side effects.

IFN α inducible protein 6 (IFI6) is an IFN-stimulated gene, belongs to the FAM14 family of the gene that maps to chromosome 1P35 (19–21). *In Silico* analysis has identified four FAM14 genes in humans (IFI6, IFI27, ISG12*b*, and ISG12*c*) and three in mice (ISG12*a*, ISG12*b1*, and ISG12*b2*) (20). However, the function of these genes has not been fully characterized, and their physiological role is still elusive. Based on a sequence homology induced by type I IFN, the FAM14 family contains two prominent genes, IFI6 and IFI27, both are considered to be a mitochondrial proteins with a different function in apoptosis but the precise localization is still not clear. Both IFI6 and IFI27 are small hydrophobic protein shared 36% overall amino acid sequence. The IFI6 stabilizes the mitochondrial function that leads to discontinuation of apoptosis whereas the expression of IFI27 destabilizes the mitochondrial function and leads to apoptosis (22). The IFI6 is a 13 kDa protein of 130 amino acids that plays a critical role in immunomodulation and an antiapoptotic function by activation of JAK/STAT signaling pathway that subsequently blocks the mitochondrial release of cytochrome c and discontinues the apoptosis (23).IFI6 is a pro-survival protein that antagonizes the TRAIL (tumor necrosis factor related apoptosis inducing ligand) induced apoptosis in human myeloma cell through inhibiting the intrinsic apoptotic pathway (24). These studies demonstrated that the FAM14 family has diverse biological function, depending on the cell/tissue type they reside. The type I IFN is highly induced the expression of IFI6. A study based on the interaction of hepatoma cells with HBV suggested that the downregulation of HBV in hepatoma cells with siRNA changed the expression profile of some of the IFN-stimulated genes (IFI6, MDA5, IFI27, STAT1, IFIT1, ISGF3G, IFITM1, OAS1, and G1P2). These genes might be responsible for the interaction of HBV with host cells (25). A previous study on IFI6 indicates the association of IFI6 polymorphism with the chronicity of the chronic liver disease. These polymorphisms in IFI6 lead to modulate the IFI6 expression level and delay the release of cytochrome c that eventually block the apoptotic signal through HBV specific CD8+ T cells. After escaping from antigen-induced apoptosis, CD8+ T cells proliferate and differentiate into activated CD8+ T cells to clear HBV infection (26). However, only a few studies have been conducted to demonstrate the antiviral activity of IFI6. Emerging evidence on IFI6 strongly proposed its association with the immune system but the functional and antiviral role of IFI6 is not fully elucidated in HBV replication and gene expression.

In this study, we hypothesized the functional and antiviral role of IFI6 against HBV replication and gene expression. The overexpression and knockdown inhibit and enhances HBV replication and transcription, respectively. The *in vitro* study of IFI6 indicates a putative binding site of HBV that could restrict HBV EnhII/Cp promoter activity while *in vivo* study demonstrated a substantial reduction of HBV replication and gene expression.

## Materials and Methods

### Ethics Statement

All mice were kept in a pathogen-free animal facility at Wuhan University. All experiments were performed per the instructions of the of Health National Institutes Guide for the Care and Use of Laboratory Animals. The protocol was approved by the Institutional Animal Care and Use Committee of Wuhan University (Project License WDSKY0201302 and WDSKY0201802).

### Plasmids Construction

The pHBV1.3 plasmid (HBV, genotype D, GenBank accession number V01460.1) and the reporter plasmids were constructed as previously described (27). Briefly, the EnhI/Xp-Luc (950-1375 nt), EnhII/Cp-Luc (1415-1815 nt), SP1-Luc (2707-2849 nt), and SP2-Luc (2937-3182 nt) DNA fragments and their truncation mutants were amplified by PCR and cloned into pGL3-Basic (Promega, Madison, WI) at the HindIII and SacI restriction sites. The HA-tagged IFI6 (N-terminally tagged) fragment was amplified by PCR and introduced in the pCAGGS vector (Invitrogen, Carlsbad, CA). The pLKO.1 vector was used for insertion of Control short hairpin RNA (shRNAs) targeting GFP (enhanced green fluorescence protein) and Two IFI6 short hairpin RNAs. The shRNAs target sequence is shRNA (cont.) 5’- GCAGAAGAACGGCATCAAG-3’, shIFI6-1 5’- CCTCCCAAGTAGGATTA-3’, and shIFI6-2 5’- TCCAGAACTTTGTCTAT-3’.

### Cell Culture and Transfection

HepG2, Huh7, and HEK293T cells were maintained in DMEM (Dulbecco’s modified Eagle’s medium) supplemented with 10% fetal bovine serum, 100 µg/ml streptomycin, and 100 U/ml penicillin. Transfection of HepG2 cells was carried out with Lipofectamine 3000 (Invitrogen) according to the manufacturer’s instructions.

### Lentiviral Production

The production of lentivirus for short hairpin RNAs was performed as described previously (28). Briefly, HEK293T cells (in a 10-cm cell culture dish) were cotransfected with 2 μg shRNA (cont.) shIFI6-1 and shIFI6-2 (cloned into pLKO.1 shRNAs vector), 1.5 μg pMD2.G, and 1.5 μg psPAX with neofect transfection reagents (Neofect Biotech, Beijing, China) according to manufacturer instructions. Supernatant from cells was collected at 48 hours and 72 hours post-transfection, and the lentivirus was centrifuged, filtered, and subsequently preserved at -80°C. Lentiviruses were used to infect HepG2 cells twice to increase transduction efficiency. The 2 μg/ml puromycin (NIH3T3) was added to cultivate knockdown cell lines.

### Enzyme-Linked Immunosorbent Assay (ELISA)

Secreted proteins (HBsAg and HBeAg) from cell culture supernatant and mice serum were detected by ELISA kit (Kehua Bio-Engineering, Shanghai, China) according to manufacturer instructions at indicated time points. The activity of β-galactosidase was used to normalize the values in cell lysates and measured by a Beta-Glo kit (Promega).

### Western Blot

Cells were washed with cold PBS, lysed with lysis buffer containing 25 mM tris-HCL (pH 7.5), 150 mM NaCl, 1 mM EDTA, 1% Triton X-100, mixed with 5% SDS loading buffer, and heated for 5 minutes. The samples were electrophoresed with SDS PAGE and subsequently transferred to nitrocellulose membrane (GE healthcare). The membrane was blocked with 5% skim milk having Tween 20 (0.1%) and probed overnight with antibodies IFI6 (Abclonal, Cat No, A6157), HA-tag (abcam, Cat No. ab9110), beta-actin (abClonal, Cat No, AC028). The signals were detected with an enhanced chemiluminescence (ECL) substrate (Millipore, Billerica, MA).

### Cell Cytotoxicity Assay

HepG2 cells were seeded in 96 well cell culture plates for 24 hours and co-transfected with the indicated amount of plasmids. The shRNA mediated knockdown cells stably expressing shGFP, shIFI6-1 and shIFI6-2 were seeded in 96 well cell culture plate for 24 hours and transfected with pHBV1.3 (80 ng) and pSV-β-gal (20 ng). Cell toxicity was measured after 48 hours of transfection with the CCK8 kit (Dojindo) according to the manufacturer’s instructions.

### Nuclear and Cytoplasmic Protein Extraction

A nuclear and cytoplasmic protein extraction kit (Applygen) was used for the extraction of nuclear and cytoplasmic IFI6 protein according to the manufacturer’s instructions.

### RNA Extraction From Cells and Liver Tissue of Mice

Total RNA from cells and tissue from mice liver was extracted by Trizol reagent according to the manufacturer’s instructions. Reverse transcription (RT-PCR) of total RNA into cDNA was performed with the PrimeScript RT reagent kit (Takara). The qRT-PCR was performed to amplify cDNA using SYBR Green Fast qPCR Master Mix (Cat No. 11201-11203: Yeasen, China). To standardize the samples with human and mouse GAPDH, the ΔΔCt method was used, and the primer sequence is mentioned in **Supplementary Table 1**.

### HBV Transcripts Analysis With Northern Blot

HBV transcripts were analyzed by northern blot as previously described (27). Briefly, HBV RNA from transfected cells was extracted and purified with Trizol Reagent (Invitrogen) according to manufacturer instructions. The 4 µg from purified RNA were resolved on a 1.5% MOPS agarose gel containing 2.2 M formaldehyde, transferred onto a nylon membrane (GE Healthcare), immobilized with UV crosslinking, HBV transcripts corresponding to nucleotides 1072-2171 of HBV genome were detected with a DIG-labeled RNA probe. Dig Northern Starter Kit (Roche Diagnostics) was used for the preparation of probe and membrane detection. The quantity of 18S and 28S rRNAs were used as internal loading controls.

### Analysis of Reporter Activity With Dual-Luciferase Reporter Assay

Dual-Luciferase Assay was carried out to determine HBV reporter activity in HepG2 cells as previously described (27). Cells were seeded in 24 well cell culture plates, and after 24 hours of seeding, cells were transfected with HBV reporters (200 ng) along with an indicated quantity of control vector (pCAGGS) or IFI6 expression plasmid (250 ng). The pRL-TK (50 ng) plasmid was added as a transfection efficiency control. After 48 hours of transfection, the cells were washed and lysed with PLB (passive lysis buffer) and subjected to luciferase activity assay using the Dual-Glo system (Promega, Madison, WI).

### Extracellular Encapsidated and Core Associated HBV DNA Analysis

The extracellular encapsidated DNA from the supernatant and mice sera were extracted and purified as previously described (29). Briefly, 80 µl of supernatant and 10 µl of mice sera were digested with 10 mM MgCl and DNase I to remove plasmid and free DNA. The enzymes were inactivated with 10 mM EDTA for 15 min at 70°C, and the samples were transferred into a lysis buffer containing 20 mM Tris-HCl, 50 mM NaCl, 20 mM EDTA, and 0.5% sodium dodecyl sulfate (SDS) containing 27 µg proteinase K, followed by overnight incubation at 65°C. Extraction of HBV DNA was performed with traditional phenol-chloroform extraction, followed by ethanol precipitation.

To extract core associated HBV DNA, the HepG2 cells were transfected with pSV-β-gal, pHBV1.3, and control vector (pCAGGS) or IFI6 in the indicated amount. After 96 hours of transfection, cells were washed and lysed in NP-40 lysis buffer containing 50 mM Tris-HCl (pH 7.4), 1 mM EDTA, and 1% NP-40 at 4°C for 30 min and centrifuged. The β-galactosidase activity assay was carried out as a transfection efficiency control in the same lysate. After centrifugation, the supernatants were collected and digested at 37°C for 1 hour with DNase I (Thermoscientific) then the enzymes were inactivated with 10 mM EDTA for 15 min at 70°C. The core-associated HBV DNA was purified with proteinase K digestion followed by traditional phenol-chloroform extraction and ethanol precipitation. The extracted DNA was subjected to qPCR using the primer RCCCS (nt 256-274) and RCCCAS (nt 421-404) (30). The primer sequence is mentioned in the **Supplementary Table 1**.

### ChIP Assay

ChIP assay was carried out as described previously (31) with a ChIP Assay kit (Beyotime, catalog number P2078) according to the manufacturer’s instructions. Briefly, HepG2 cells were seeded into a 10 cm cell culture dish and transfected with pHBV1.3 (5 µg) with IFI6 (5 µg) or control vector (pCAGGS, 5 µg). At 48 hours post-transfection, cells were crossed linked with 1% formaldehyde at 37°C for 10 minutes, neutralized with glycine (125 mM) followed by lysis with SDS lysis buffer containing 50 mM Tris-HCl, (pH 8.0), 150 mM NaCl, 10 mM EDTA, 1% SDS along with PIC (proteinase inhibitor cocktail) on ice. The lysate was subjected to sonication on ice with three pulses for 15s at 30% power to generate a genome size of 200-1000 bp. Chromatin was precipitated with HA-IFI6 antibody (abcam, Cat No, ab9110) at 4°C overnight along with Anti-RNA Polymerase II and normal mouse IgG separately as a positive and negative control, respectively. The precipitate was then washed, and immunoprecipitated DNA fragments were extracted with phenol-chloroform extraction. The DNA fragments were amplified with PCR using EnhII/Cp 1715-1815 nt primers mentioned in **Supplementary Table 1**.

### Recombinant IFI6 Protein Purification

*Escherichia coli* BL21 (Invitrogen) was used to express GST-IFI6 protein at 16°C. The protein expression was induced in 0.7 mM IPTG (isopropyl-β D thiogalactopyranoside) and cells were collected after 12 hours of induction. Lysis buffer containing 50 mM Tris-HCl (pH 7.6), 1 mM EDTA, 150 mM NaCl, 0.1 mg/ml lysozyme, 1 mM DTT (dithiothreitol), and 0.05% NP-40 were used to lyse the cells. The recombinant protein was purified with GST-Fast Flow according to the manufacturer’s instructions. The recombinant protein was quantified and stored in glycine at -80˚C.

### Electrophoretic Mobility Shift Assay

The GST-IFI6 protein was mixed in a binding buffer containing 400 mM Tris-HCl [pH 8.0], 50 mM DTT) and incubated for 30 minutes on ice as previously described (31). The CY5-labeled probe of the HBV EnhII 1715-1770 nt and non-competitive sequence (probe sequence is shown in **Supplementary Table 2**) was added to the protein mix and incubated for 30 minutes at room temperature. The samples were electrophoresed in 1% agarose gel for 1 hour at 130V and exposed to the gel for analysis with Typhoon FLA9500 (GE).

### *In Vivo* Transfection

Hydrodynamic based tail vein injection in mice was used to transfect plasmids. Briefly, pSV-β-Gal (5µg) and pHBV 1.3 (10 µg) along with control vector (20 µg) or IFI6 (20 µg) mixed in saline solution and introduced into the tail vein of eight weeks old C57BL/6 male mice (5 mice in each group) within 5-8 seconds in a volume of saline equivalent to 10% body weight of the mouse. Five days post-injection, mice were slaughtered, and serum was collected to analyze HBV DNA, HBsAg, and HBeAg. For analysis of IFI6 and HA-tag protein, western blot was performed with a small piece of mice liver, homogenized in RIPA lysis buffer along with a phosphatase inhibitor cocktail (PIC). The 10 µg of protein samples were resolved in SDS-PAGE and analyzed with enhanced chemiluminescence (ECL) substrate (Millipore, Billerica, MA). For HBV RNA analysis, a small slice of mice liver was homogenized in Trizol reagent and extracted and purified total RNA. Reverse transcription (RT-PCR) of total RNA into cDNA was performed with the PrimeScript RT reagent kit (Takara). SYBR Green Fast qPCR Master Mix (Cat No. 11201-11203: Yeasen, China) was used to amplify cDNA, and qRT–PCR was performed. The primers for qRT–PCR, qPCR, and mice GAPDH are mentioned in **Supplementary Table 1**.

### Statistical Analysis

**Figure 1:**
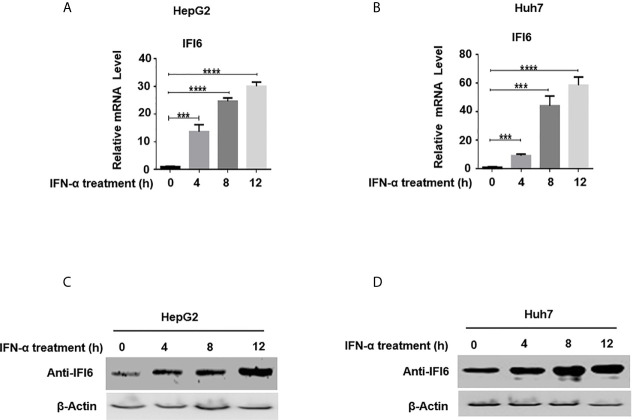
Expression level of IFI6 is significantly induced by IFN-α in Hepatocytes. HepG2 and Huh7 cells were seeded in 12 well cell culture plates. After 24 hours, cells were treated with human IFN-α (100 ng/ml). Samples were collected at 0, 4, 8, and 12 hours after IFN-α treatment and total RNA was purified and subjected to qRT-PCR **(A, B)** and western blot for protein detection **(C, D).** The anti-IFI6 antibody was used to determine the protein level of IF6. Expression of β-actin was used as a loading control. The data are averages of three independent experiments; the results were measured as a mean ± SD and statistically analyzed. ***P < 0.001, ****P < 0.0001.

**Figure 2:**
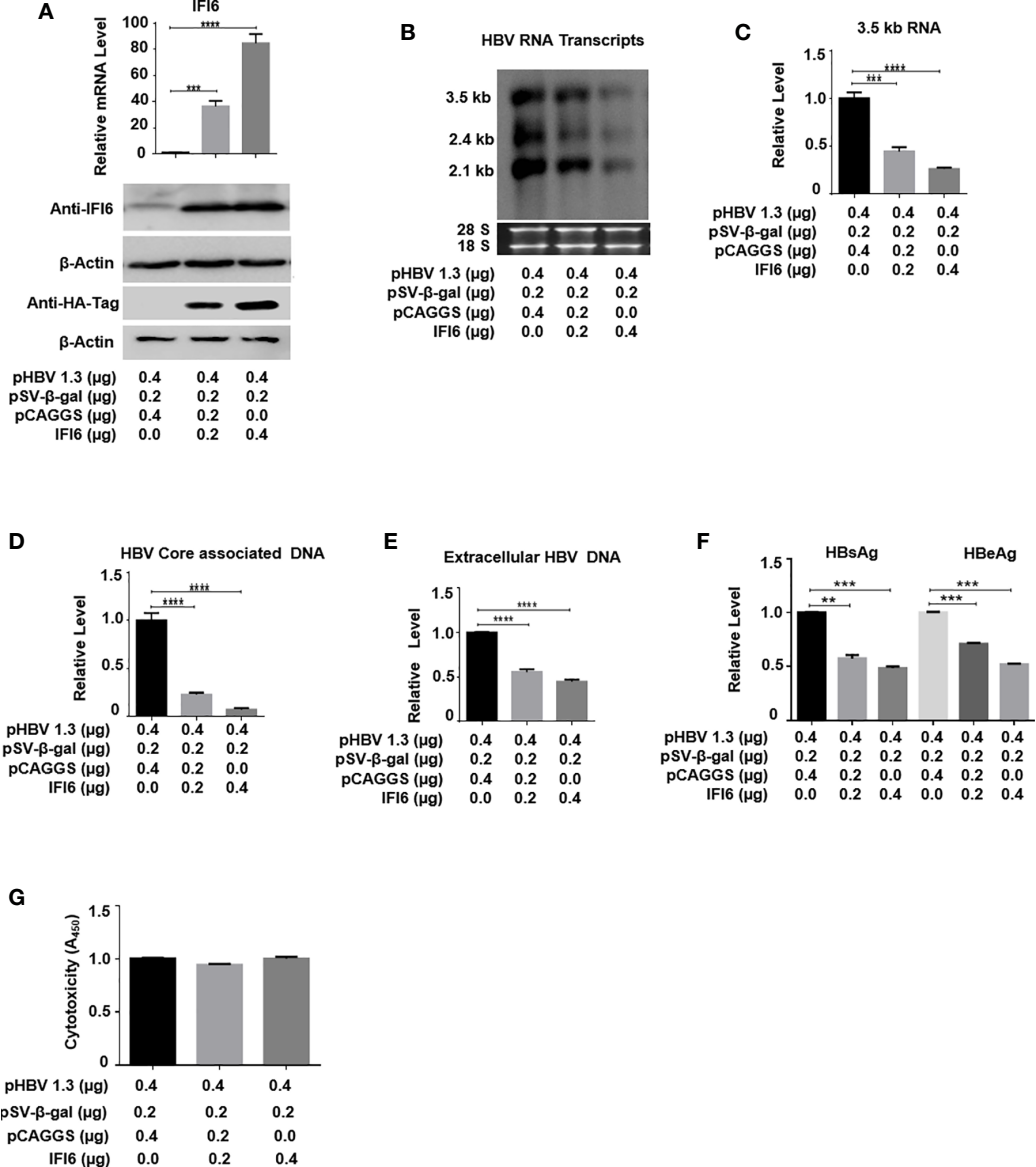
IFI6 overexpression inhibits HBV replication and gene expression. HepG2 cells were seeded in 12 well cell culture plates and transfected with indicated amounts of the plasmid. Cells were harvested after 48 h posttransfection **(A, B, C, F, G)** or 96 hours post-transfection **(D, E). (A)** The expression of IFI6 mRNA and protein was measured by qRT-PCR and western blot, respectively. The anti-IFI6 and anti-HA-tag antibodies were used to determine the protein level of IF6 and HA-tag, respectively. The β-actin was used as a loading control. **(B)** HBV RNA transcripts (3.5 kb, 2.4 kb, and 2.1 kb) were determined with northern blot. The amount of 28S and 18S rRNAs was used as a loading control. **(C)** The level of HBV 3.5 kb RNA was measured with qRT-PCR. The GAPDH level was used as an internal control. HBV core associated **(D)** and extracellular DNA **(E)** was determined by qPCR. **(F)** The level of secreted HBsAg and HBeAg from transfected cell supernatant was measured with ELISA. **(G)** The cytotoxicity of transfected HepG2 was determined with a CCK8 kit after 48 hours of transfection with the indicated amount of plasmids. The data are averages of three independent experiments; the results were measured as a mean ± SD and were statistically measured. *** *P* < 0.001, **** *P* < 0.0001.

**Figure 3:**
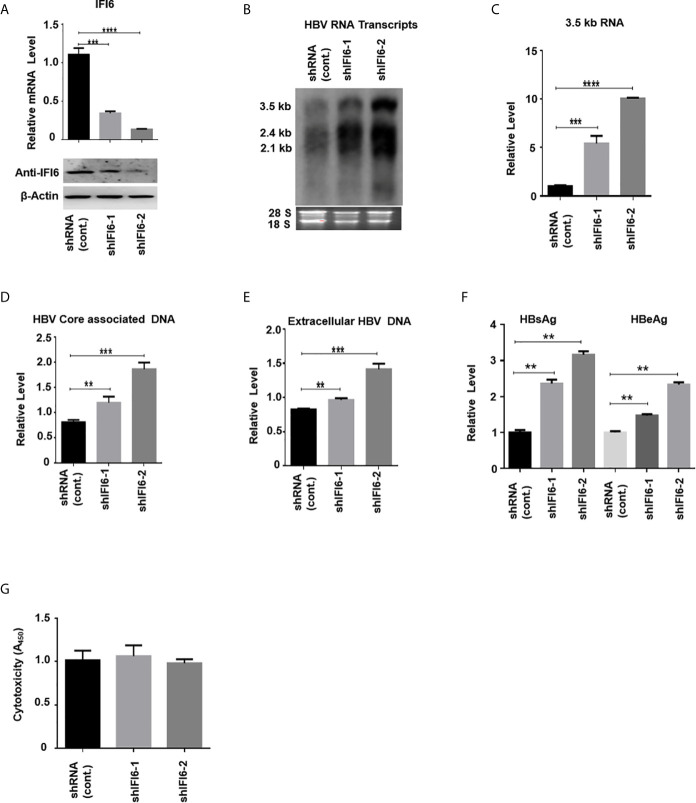
Knockdown of IFI6 enhances HBV replication and gene expression. Stable HepG2 cell lines expressing shRNA (control), shIFI6-1, and shIFI6-2 were seeded in 12 well cell culture plate and transfected after 24 hours with pHBV1.3 (800 ng) and β-Gal (200 ng) and cells were harvested 48 hours posttransfection **(A, B, C, F, G)** or 96 hours posttransfection **(D, E)**. **(A)** The IFI6 mRNA and the protein expression level were measured by qRT-PCR and western blot, respectively. The anti-IFI6 antibody was used to determine the protein level of IF6. The β-actin was used as a loading control. **(B)** HBV RNA transcripts were determined with northern blot. The amount of 28S and 18S rRNAs was used as a loading control. **(C)** The level of HBV 3.5 kb RNA was measured with qRT-PCR. The GAPDH level was used as an internal control. **(D)** HBV core associated DNA and extracellular DNA **(E)** was determined with qPCR. **(F)** The level of secreted HBsAg and HBeAg from transfected cell supernatant was measured with ELISA. **(G)** The cytotoxicity of the stable knockdown cell line was determined with CCK8 kit after 48 hours of transfection with pHBV (800 ng) and β-Gal (200 ng). The data are averages of three independent experiments; the results were measured as a mean ± SD and were statistically measured. ** *P* < 0.01, *** *P* < 0.001, **** *P* < 0.0001.

**Figure 4:**
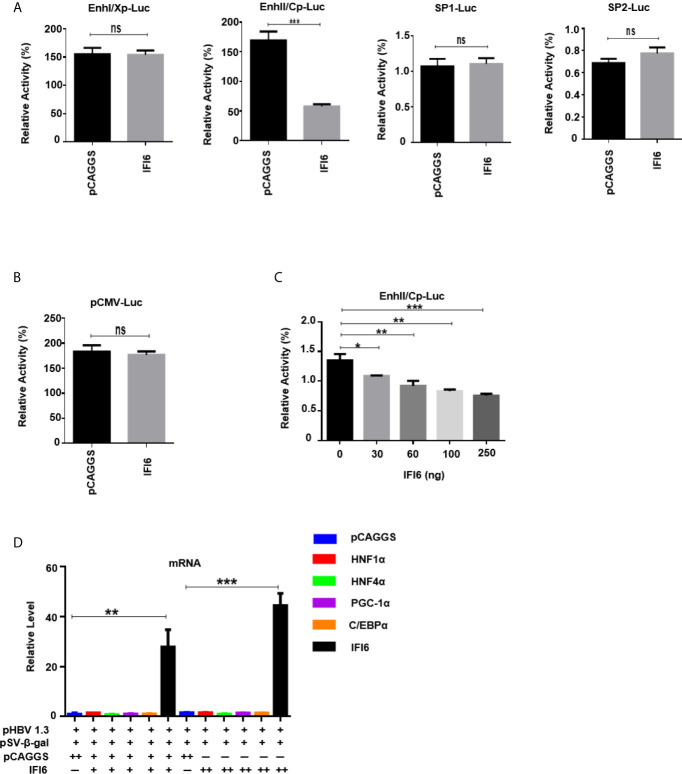
IFI6 downregulate HBV gene expression by suppressing EnhII/Cp promoter activity. HepG2 cells, along with IFI6 expression plasmid or control plasmid (250 ng) was transiently transfected with four reporter plasmids (200 ng) in 24 well cell culture plates and harvested the cells after 48 hours of transfection and subjected to Dual-Luciferase assay. **(A)** The activity of four promoters was measured, and the pRL-TK (50 ng) was used as a transfection efficiency control. **(B)** The activity of the pCMV promoter (200 ng) was measured with a Dual-Luciferase assay. **(C)** The activity of EnhII/Cp was measured in the indicated dose. **(D)** The level of different liver enriched transcription factors was measured by qRT-PCR. The + in pHBV1.3 showing 0.4 µg concentration, the + in pSV-β-gal showing 0.2 µg concentration, the + and ++ in pCAGGS and IFI6 indicate 0.2 and 0.4 µg concentration of pCAGGS and IFI6, respectively, and - in pCAGGS and IFI6 panel is showing zero concentration of pCAGGS and IFI6. The data are averages of three independent experiments; the results were measured as a mean ± SD and were statistically measured. *p < 0.05, **p < 0.01, ***P < 0.001, ns, non-significant.

**Figure 5:**
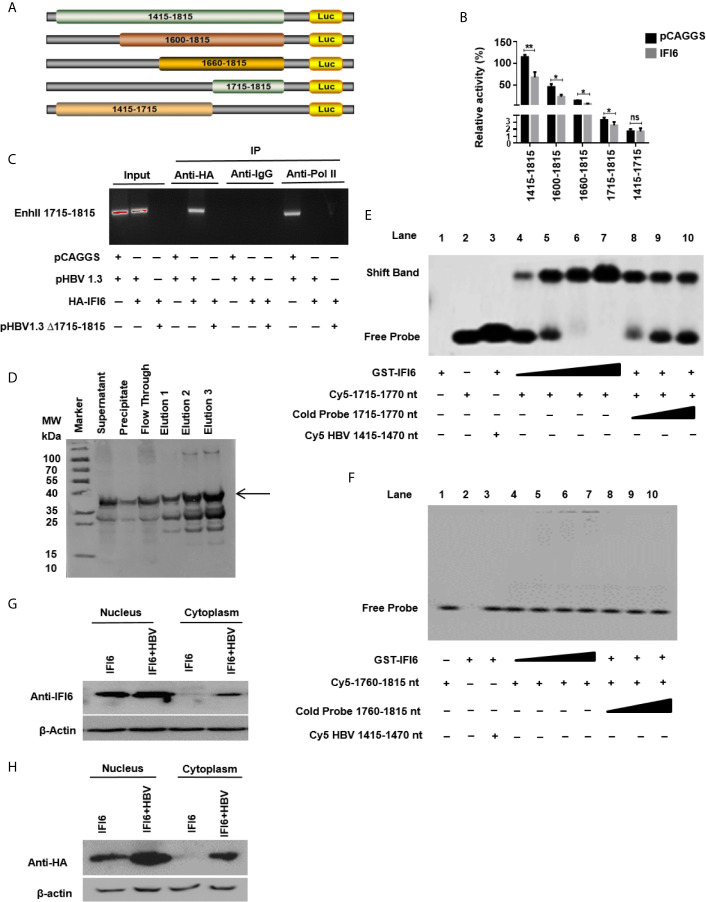
IFI6 suppresses HBV replication by binding to 1715-1770 nt of EnhII/Cp promoter. **(A)** Schematic diagram of EnhII/Cp promoters and its truncated mutants. **(B)** The mutants were subcloned into pGL3 basic plasmid and transfected in HepG2 cells. Cells were harvested after 48 h of transfection, and Dual-luciferase activity was measured. **(C)** HepG2 cells were seeded in a 10 cm dish and transfected with pHBV1.3 (5 µg) with IFI6 (5 µg) or control plasmid (pCAGGS, 5 µg), after 48 hours of transfection, cells were harvested, immunoprecipitate was extracted with overnight incubation of Anti-Polymerase II, mouse IgG, and Anti-HA-Tag antibody to analyze the binding ability of EnhII 1715-1815. The protein-bound EnhII 1715-1815 DNA was extracted, amplified with PCR, and detected with agarose gel electrophoresis. **(D)** Expression and purification of recombinant GST-IFI6 protein were performed from *E.coli*. The western blot was performed to measure the size of the GST-tagged IFI6 protein. The arrow indicated the expected band size of protein, and elution 3 was used for subsequent EMSA experiments. **(E)** EMSA was performed to determine the specific binding of IFI6 with EnhII/Cp 1715-1770 region. Lane 1, GST IFI6 protein alone, Lane 2, Cy5 labeled probe (1715-1770 nt) alone, Lane 3, Cy5 labeled probe (1715-1770 nt) incubated with non-specific competitor probe (Cy5 1415-1470), Lane 4-7, Cy5 labeled probe (1715-1770 nt) incubated with different concentrations of GST IFI6 protein, Lane 8-10, a competition of Cy5 labeled probe (1715-1770 nt) with different concentrations of an unlabeled cold probe (1715-1770 nt). **(F)** EMSA was used to determine the binding of IFI6 with EnhII/Cp 1760-1815 region. Lane 1, Cy5 labeled probe (1760-1815) alone, Lane 2, GST IFI6 protein alone, Lane 3, Cy5 labeled probe (1760-1815 nt) incubated with non-specific competitor probe (Cy5 1415-1470), Lane 4-7, Cy5 labeled probe (1760-1815 nt) incubated with different concentrations of GST IFI6 protein, Lane 8-10, a competition of Cy5 labeled probe (1760-1815 nt) with different concentrations of an unlabeled cold probe (1760-1815 nt). **(G)** HepG2 cells were seeded in 12 well cell culture plates and transfected after 24 hours with IFI6 (1 µg) alone or co-transfected with pHBV1.3 (0.5 µg). Cells were harvested after 48 h posttransfection. Nuclear and cytoplasmic protein was extracted and subjected to western blot. The anti-IFI6 and **(H)** anti-HA-tag antibodies were used to determine the protein level of IF6 and HA-tag respectively from nuclear and cytoplasmic extracted protein. The β-actin was used as a loading control. The data shown in the graph are averages of three independent experiments; the results were measured as a mean ± SD and were statistically measured. * *P* < 0.05, ** *P* < 0.01, ns, non-significant.

**Figure 6:**
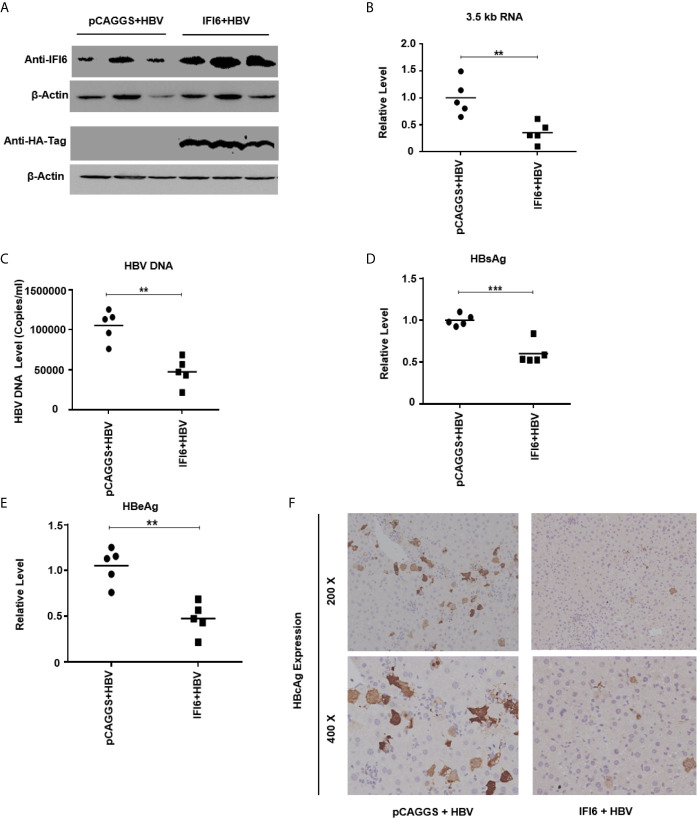
IFI6 inhibits replication of HBV and gene expression in mice. The IFI6 (20 µg) or its control vector (pCAGGS, 20 µg) and pHBV1.3 (10 µg) plasmid were hydrodynamically injected in eight weeks old male C57BL/6 mice. After 5 days post-injection, mice sera and liver were collected. **(A)** The protein was extracted from mice liver and expression of IFI6 and HA-tag protein was determined with western blot using anti-IFI6 and anti-HA-tag antibody. The β-actin was used as a loading control. **(B)** The level of 3.5 kb HBV RNA was measured by qRT-PCR. The level of mice GAPDH was used as an internal control. **(C)** The HBV DNA was extracted from mice serum, and the level was determined by qPCR. The secreted HBsAg **(D)** and HBeAg **(E)** were determined with the ELISA. **(F)** The immunohistochemical staining of HBcAg from mice liver. The data are averages of three independent experiments; the results were measured as a mean ± SD and were statistically measured. ** *P* < 0.01, *** *P* < 0.001.

All experiments were performed at least three times and the results were measured as a mean ± SD. An independent Student’s *t*-test was used to measure the statistical differences between normally distributed samples (**Figures 1A**, **3C, D, F**, **4A–D**, **6B–E**) and non-parametric Student’s *t*-test was used for non-normally distributed samples (**Figures 1B**, **2C–F**, **3E**, **5B**) by using GraphPad Prism 6.01 (GraphPad Software Inc, USA). A *p* < 0.05 was considered statistically significant.

## Results

### Expression of IFI6 Is Induced by IFN-α in Hepatocytes

IFN-α activates a cascade of the JAK/STAT pathway upon binding to the IFN receptors and subsequently triggers hundreds of IFN-stimulated genes (ISGs). IFI6 is one of the IFN stimulated genes (32). We first determined the IFI6 expression level by inducing the cells with IFN-α and assessed the mRNA level of IFI6 by qRT-PCR. The IFI6 expression was upregulated in a dose-dependent manner in HepG2 and Huh7 cells by the induction of IFN-α (**Figures 1A, B**). Furthermore, IFI6 protein was highly expressed in both types of cell lines by IFN-α stimulation (**Figures 1C, D**). The expression level of IFI6 by stimulation of IFN-α indicates a unique role of IFI6 against foreign invaders in terms of host defense strategy in hepatocytes.

### IFI6 Overexpression Inhibits HBV Replication and Gene Expression

As the function of IFI6 on HBV replication and gene expression was not reported previously, we initially evaluated the role and function of IFI6 in HBV replication and gene expression in the HepG2 cell line. HA-tagged IFI6 or control vector (pCAGGS) at different concentrations was transiently transfected along with β-gal and pHBV1.3 in HepG2 cells. The mRNA and protein levels of IFI6 were detected with qRT-PCR and western blot, respectively. The IFI6 was highly expressed in a dose-dependent manner in HepG2 cells (**Figure 2A**). As the transcription of HBV RNA is controlled by core promoter and EnhII, we further evaluated the level of HBV RNA transcripts (3.5 kb, 2.4 kb, and 2.1 kb) by northern blot. The results indicated that the steady-state level of 3.5 kb pgRNA along with 2.4 kb, and 2.1 kb RNA transcripts were decreased in a dose-dependent manner (**Figure 2B**). Consistent with decreased HBV transcripts, the qRT-PCR analysis indicated the reduction of 3.5 kb HBV RNA level by overexpression of IFI6 (**Figure 2C**). As 3.5 kb HBV RNA acts as a template for HBV DNA replication, we further confirmed the HBV DNA replication inhibition in HepG2 cells and measured the level of HBV intracellular core-associated and extracellular encapsidated DNA level by q-PCR. The intracellular core-associated and extracellular HBV DNA replication significantly reduced upon overexpression of IFI6 (**Figures 2D,E**). To determine the HBV secreted proteins (HBsAg and HBeAg) level in supernatant of transfected cells, ELISA was performed, and the level of HBsAg and HBeAg in cell supernatant was highly reduced, indicating the inhibitory role of IFI6 (**Figure 2F**). Furthermore, we determined the cytotoxicity of transfected HepG2 cells with CCk8 assay. These results demonstrated that transfection of HepG2 cells with IFI6 plasmid has no cytotoxic effect in our experimental system (**Figure 2G**). Taken together, we conclude that IFI6 overexpression inhibits HBV replication and gene expression in HepG2 cells.

### Knockdown of IFI6 Enhances HBV Replication and Gene Expression

The above data indicate that overexpression of IFI6 inhibits the replication of HBV and gene expression. To further evaluate the antiviral activity of IFI6 against HBV, we investigated whether downregulating the activity of IFI6 could enhance HBV gene expression. We used short hairpin RNA targeting two different regions of IFI6 and inserted it into the cell genome by lentivirus infection. We generated three stable HepG2 cell lines expressing control (shRNA) and two different target regions of IFI6 (named shIFI6-1 and shIFI6-2). The downregulating efficiency of stable cell lines was measured by qRT-PCR and western blot, respectively. The level of IFI6 mRNA and protein was significantly reduced as compared with control (**Figure 3A**). To further confirm the activity of stable IFI6 cell lines with HBV gene expression, pHBV1.3 and β-gal were transiently transfected and measured the level of HBV transcripts (3.5 kb, 2.4 kb, and 2.1 kb) by northern blot. The steady-state level of HBV RNA transcripts (3.5 kb, 2.4 kb, and 2.1 kb) was enhanced significantly by the downregulation of IFI6 activity (**Figure 3B**). Accordingly, the qRT-PCR analysis indicated the elevated level of 3.5 kb HBV RNA by IFI6 knockdown (**Figure 3C**). Consistent with the increased HBV RNA transcripts level, the level of HBV intracellular core associated and extracellular DNA level was measured with q-PCR, and the replication of HBV DNA was increased potently (**Figures 3D, E**). Moreover, the level of secreted HBsAg and HBeAg in the cell culture supernatant was significantly increased (**Figure 3F**). However, to determine the side effect of shRNA on HepG2 cells, cytotoxicity assay was performed with CCK8 kit, indicating no side effect of shRNA on growth condition of stable knockdown cell lines (**Figure 3G**). These results showed that the HBV gene expression increased by downregulating the activity of IFI6.

### IFI6 Downregulate HBV Gene Expression by Suppressing ENHII/Cp Promoter Activity

The HBV transcription is controlled by different promoters EnhI, EnhII, Sp1, and Sp2. To further evaluate the molecular mechanism of IFI6 mediated HBV suppression and measured the HBV promoters activity, the EnhI, EnhII, Sp1, and Sp2 were cloned into pGL3 basic vector to construct four reporter plasmids. HepG2 cells along with IFI6 expression plasmid or control plasmid was transiently transfected with four reporter plasmids and measured the dual-luciferase activity. The EnhII/Cp was significantly suppressed by overexpression of IFI6 (**Figure 4A**) while all other promoters were not affected by IFI6 overexpression. To further clarify that whether inhibition of EnhII/Cp is specifically due to overexpression of IFI6, we replaced EnhII/Cp with pCMV (cytomegalovirus promoter) and HepG2 cells along with IFI6 expression plasmid or control plasmid were transiently transfected with pCMV construct and measured the dual-luciferase activity. Interestingly, IFI6 does not change the activity of the pCMV promoter, indicating the specificity of IFI6 mediated inhibition of EnhII/Cp (**Figure 4B**). Moreover, dose-dependent of IFI6 significantly decreased the activity of EnhII/Cp promoter (**Figure 4C**). These results indicated that IFI6 downregulates HBV at the transcriptional level. Several previous studies revealed that liver enriched transcription factors such as HNF1α, HNF4α, PGC-1α, and C/EBPα are responsible for regulating the Enhancer activity of HBV (33–36). As the IFI6 inhibits the activity of EnhII/Cp, we further investigated the role of these liver enriched transcription factors and analyzed their activity by qRT-PCR. The overexpression of IFI6 did not alter the activity of these transcription factors (**Figure 4D**) suggesting that IFI6 has a direct influence on HBV EnhII/Cp activity. Taken together, our results indicated that IFI6 directly suppresses EnhII/Cp activity

### IFI6 Suppresses HBV Replication by Binding to 1715-1770 nt of EnhII/Cp Promoter

As IFI6 directly suppresses the EnhII/Cp promoter activity, we further evaluated the specific binding site of the EnhII/Cp promoter. To investigate the specific binding site of EnhII/Cp promoter, a series of EnhII/Cp deletion mutant was generated and cloned into pGL3 basic vector to construct five reporters (**Figure 5A**). HepG2 cells along with IFI6 expression plasmid or control plasmid was transiently transfected with five reporters and dual-luciferase activity was measured. The IFI6 reduced the activity of EnhII/Cp 1715-1815 bp (100 bps) (**Figure 5B**). To further identify the 100 bp binding site of EnhII/Cp, chromatin immunoprecipitation assay (ChIP) was performed. ChIP assay indicated that IFI6 specifically binds to 1715-1815 nt of EnhII/Cp, however, no binding was observed between IFI6 and EnhII/Cp Δ1715-1815 nt when 1715-1815 bp were deleted (**Figure 5C**). To further identify the specific binding site of EnhII/Cp 1715-1815 bp, we generated two 55 base pairs long overlapped Cy5 labeled probes (1715-1770 bp, 1760-1815 bp) to identify the specific binding sites by Electrophoretic mobility shift assay. Recombinant GST-IFI6 protein was expressed and purified from *E.coli* (**Figure 5D**) and EMSA was performed to detect the binding of IFI6 with EnhII/Cp 1715-1815 bp. The results indicated that IFI6 could directly bind to the EnhII/Cp 1715-1770 bp (**Figure 5E**) while no binding was observed between the IFI6 protein and EnhII/Cp 1760-1815 bp (**Figure 5F**). Taken together, our results revealed that EnhII/Cp 1715-1770 bp of HBV is involved in IFI6 mediated HBV inhibition.

### IFI6 Is Localized Mainly Inside the Nucleus

Previous studies demonstrated that IFI6 localized in mitochondria or endoplasmic reticulum inside the cells (21). As we indicated that IFI6 inhibits HBV replication and gene replication inside the nucleus (**Figures 5G, H**). We asked to evaluate the localization of IFI6. To this extent, HepG2 cells were transfected with HAtagged IFI6 alone or with pHBV1.3. After 48 hours of transfection, the nuclear and cytoplasmic protein was extracted and subjected to western blot. It is demonstrated that IFI6 is highly expressed in the nucleus rather than the cytoplasm (**Figure 5G**). Moreover, the IFI6 protein expresses more in the presence of HBV inside the nucleus indicating the expression of endogenous IFI6 as well. To further confirm this localization, a western blot was performed using HAtag antibody. **Figure 5H** shows the same localization found inside the nucleus rather than in the cytoplasm. These results clearly support our hypothesis of inhibition of HBV inside the nucleus.

### IFI6 Inhibits Replication of HBV and Gene Expression in Mice

**Figure 7:**
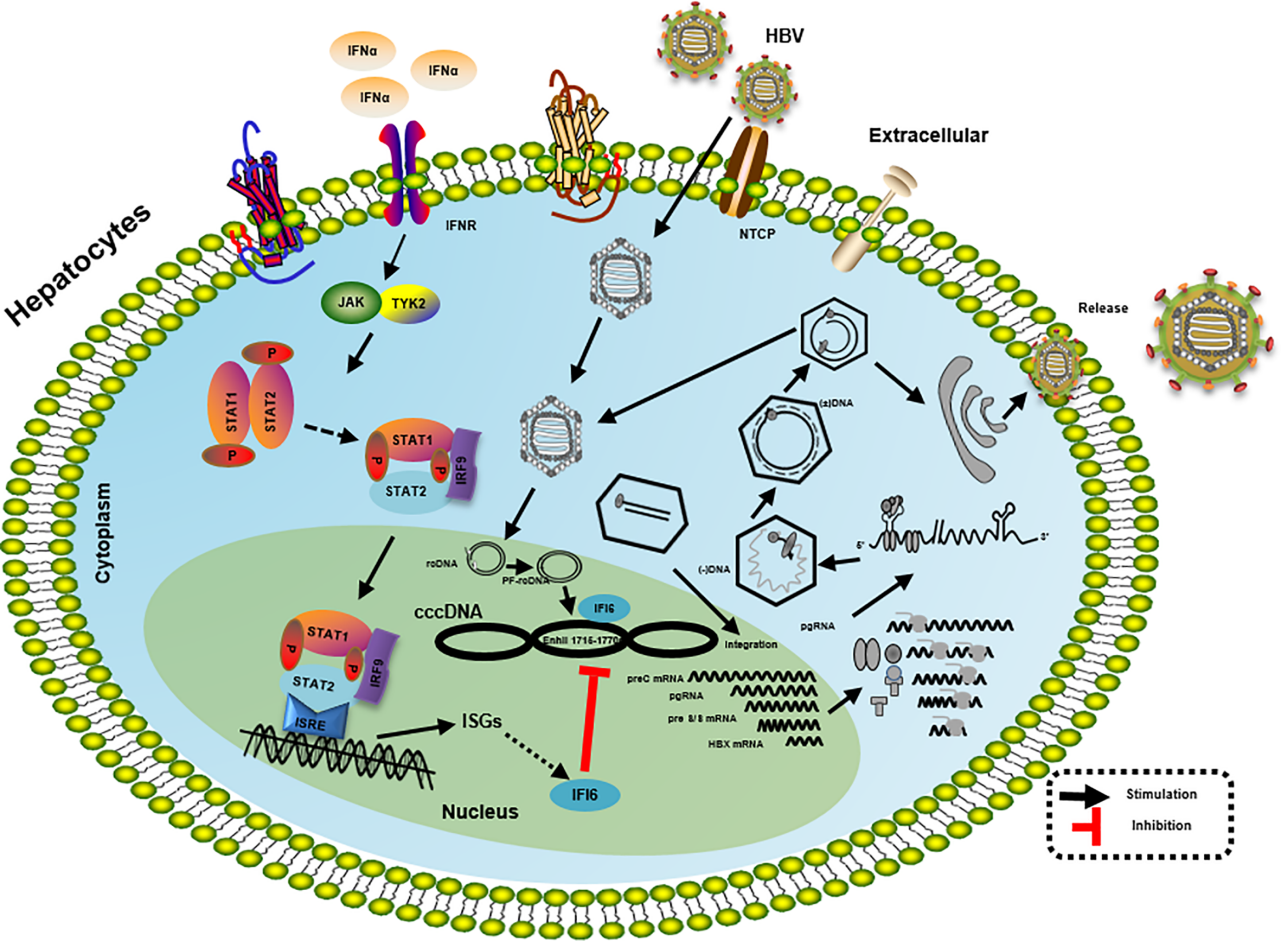
Mechanism of IFI6 mediated inhibition of HBV replication and gene expression. The type I IFN binds to the cell receptor and induces the JAK-STAT signaling pathway that subsequently induces transcription of ISGs. IFI6 is one of the IFN-stimulated genes that inhibit the transcription of 3.5 kb, 2.4 kb, and 2.1 kb RNA of HBV that subsequently inhibit the translation of different HBV proteins.

As *in vitro* results indicate that the IFI6 inhibits replication of HBV and gene expression, we further evaluated IFI6 mediated HBV inhibition in a mouse model. The IFI6 overexpression plasmid and pHBV1.3 together with its control vector were hydrodynamically injected into the tail vein of mice. After five days of injection, mice were slaughtered, liver and blood were collected. The western blot was performed to determined IFI6 and HA-tag protein levels. The results indicated a high expression of IFI6 protein level in mice liver (**Figure 6A**). To further verify the *in vivo* HBV transcription, 3.5 kb HBV RNA level was measured by qRT-PCR (**Figure 6B**), which indicated the inhibition of 3.5 kb HBV RNA which is a template of HBV transcription that subsequently leads to the inhibition of HBV DNA level (**Figure 6C**). Consistent with *in vitro* results, the level of HBV secreted HBsAg and HBeAg were significantly reduced in the mouse model (**Figures 6D, E**). To further confirm the *in vivo* inhibition of HBV, immunohistochemical staining was performed to evaluate the HBcAg level. **Figure 6F** clearly showed the reduced level of HBcAg expression level in mice liver. Taken together, our results revealed that IFI6 could inhibit the replication of HBV and gene expression *in vivo* that is consistent with *in vitro* results. Overall, our results demonstrate that induction of IFI6 inhibits HBV replication and gene expression inside the nucleus that specifically bind with the EnhII/Cp 1715-1770 nt and directly suppress the replication of HBV (**Figure 7**).

## Discussion

As HBV infection is a significant public health concern worldwide and the mechanism of HBV pathogenesis is mostly unclear. This study aims to assess the mechanism underlying the regulation of anti-HBV activity of IF6. This study identified a new horizon of a previously unidentified mechanism by which IFI6 inhibits HBV replication through specifically bind with EnhII/Cp region. The interaction between liver innate immunity and HBV infection has an essential role in HBV disease progression. During acute HBV infection, a noncytolytic mechanism triggers host T-cell responses to eliminate the virus from hepatocytes, which produces infiltrating TNF-α and IFN-γ that acts as proinflammatory antiviral cytokines (37). However, the role of IFI6 in the regulation of acute and chronic HBV infection has not been reported previously. Here we first time provide the evidence that IFI6 restricts HBV replication in *in vitro* and *in vivo*.

IFN is extensively prescribed to treat chronic HBV in clinical settings for the last couple of decades. For chronic HBV treatment, the only available immunotherapy to date is primarily based on pegylated IFN-α. However, the administration of pegylated IFN-α for HBV chronic treatment has only 20-40% seroconversion of HBeAg (38). The intracellular activity of IFN-α is primarily mediated by an array of transcription of around 300 IFN-stimulated genes (ISGs) (39). ISGs also show a tremendous antiviral effect by targeting different steps of the viral life cycle. Many of these ISG’s inhibit replication of the hepatitis C virus (40), while some of the ISG’s such as USP 18 and ISG15 enhanced HCV replication (41, 42). IFI6 is one of the IFN-stimulated genes and its antiviral mechanism and function are not fully elucidated in HBV replication. In this study, we hypothesized the functional and antiviral mechanism of the IFI6 gene against HBV gene expression and replication. In examining intrahepatic IFN-stimulated genes and evaluating the functional role of IFI6 in the context of HBV immune response, we determined the expression level of IFI6 by inducing hepatoma cell lines with IFN-α that significantly induces IFI6 expression (**Figure 1**). The overexpression of IFI6 reduced the HBV RNA transcripts and protein level, while the knocking down of IFI6 enhanced the activity of HBV gene expression and replication (**Figures 2** and **3**). A previous study based on ISG20 inhibited HBV replication in mouse hepatocytic cell lines and involved IFN-mediated HBV inhibition (43). Consistent with this study, our results of overexpression and knockdown of IFI6 significantly inhibit and elevated the replication of HBV and gene expression in HepG2 cells, respectively.

The HBV transcription is controlled by two enhancers (EnhI/Xp and EnhII/Cp) and four promoters’ elements. Previous studies revealed that IFN-α influences different steps in the HBV life cycle, including DNA replication, the formation of the core particle, transcription and degradation, and the export of viral RNAs (44–47). As the EnhII is positioned adjacent to the core promoter and regulates the transcriptional activity of precore/preS1 and preS2 promoters in an orientation independent manner. Various other cellular and transcription factors, including HNF4α, Prox1, FOXO1 bind with EnhII and promote the HBV regulation. Previous reports postulated that an ISG TRIM22 significantly suppresses HBV gene expression by targeting EnhII/Cp promoter (48). In this study, we demonstrated that IFI6 influenced the activity of EnhII/Cp while there was no effect of IFI6 on other regulatory elements of HBV (Sp1, Sp2, and EnhI) (**Figure 4A**).

HBV transcription inhibition is involved in numerous molecules of cytokines. TNF-α and IFN-γ induced IL-32 in primary human hepatocytes and hepatoma cells that regulate the transcription of HBV core promoter by downregulating HNF1-α and HNF4-α. Similarly, IFN-γ-mediated anti-HBV activity is contributed by hepatocystin that downregulates viral enhancer activity by overexpression of HNF4-α (31–34, 47, 48). According to this context, our results indicate that IFI6 inhibit HBV replication by interacting with EnhII/Cp region without dysregulation of various liver enriched transcription factors, including HNF1α, HNF4α, PGC-1α, and C/EBPα (**Figure 4C**) (34). We speculate that overexpression of IFI6 inhibits the EnhII activity but does not affect the expression level of these transcription factors. There may be some other transcription factors that could bind to the EnhII region. However, we cannot exclude the recruitments of these transcription factors in the regulation of the EnhII with IFI6 overexpression. Their activity may be changed after IFI6 binding with the EnhII. Further study will be needed to clarify this mechanism.

The regulation of HBV is determined by *cis*-acting factors within HBV mRNA and *trans*-acting factors in the host cell. We further mapped the specific sequence binding site of HBV EnhII/Cp and identified the EnhII/Cp 1715-1770 nt specific cis-acting factors in IFI6 mediated HBV inhibition in EMSA assay with a detailed mechanism (**Figure 5E**). As the overexpression and knockdown clearly indicate the anti-HBV effect of IFI6 but the level of HBsAg dramatically increased which is not under the regulation of EnhII (49). This decrease might be due to transcriptional inhibition or some other liver transcriptional factors involved. Also, the function and role of HBV were elucidated *in vivo* indicated that overexpression of IFI6 markedly inhibited the replication of HBV DNA and gene expression in mouse sera and liver (**Figure 6**).

In conclusion, our study demonstrated the functional role and antiviral effect of IFI6 on the replication of HBV DNA and gene expression *in vitro* and *in vivo*. Furthermore, we showed that IFI6 is mainly localized inside the nucleus. Collectively, our findings also provide an understanding of the antiviral mechanism of IFI6. However, the study is limited to IF6 mediated inhibition of HBV on RNA level and results are generated from *in vitro* and *in vivo* experiments where no stable cccDNA pool is detected. Further study is needed to understand the detailed mechanism and to control HBV at the cccDNA level.

## Data Availability Statement

The original contributions presented in the study are included in the article/supplementary material. Further inquiries can be directed to the corresponding author.

## Author Contributions

YC, LZ, and MS conceived the study and designed the experiments. MS, HU, and KY performed the experiments. MH, JF, MAS, RH, and QL assisted with the experiments. MS and HU analyzed the data. MS wrote the initial draft of the manuscript. DG and YC provided technical discussion. LZ revised the manuscript. All authors contributed to the article and approved the submitted version.

## Funding

This work was supported by the Fundamental Research Funds of Health Planning Committee of Hubei Province (WJ2019Q040) to LZ and China NSFC projects (81672008), Hubei Natural Science Foundation (2018CFA035) and Basic Scientific Research Foundation of Central Universities (2042019gf0026) to YC.

## Conflict of Interest

The authors declare that the research was conducted in the absence of any commercial or financial relationships that could be construed as a potential conflict of interest.
