# Supplementary material for: The Functional and Antiviral Activity of Interferon Alpha-Inducible IFI6 Against Hepatitis B Virus Replication and Gene Expression
Source: Front Immunol. 2021 Apr 1;12:634937. doi: 10.3389/fimmu.2021.634937 (PMC8047077; doi:10.3389/fimmu.2021.634937)
Supplement: Supplementary file 1 [file Table_1.docx]

Supplementary Table

**Table 1: The sequence of primers used in this study**

| **Name** | **Sequence (5’-3’)** | **use** |
| --- | --- | --- |
| IFI6 - F | CCGGAATTCGCCACCATGCGGCAGAAG | Cloning PCR |
| IFI6 - R | CCGCTCGAGCTCCTCATCCTCCTCAC |  |
| RCCCS | CTCGTGGTGGACTTCTCTC | qPCR |
| RCCCAS | CTGCAGGATGAAGAGGAA |  |
| pGL3 1415-1815F | CGAGCTCGCCACCGACGTCCTTTGTTT | Cloning PCR |
| pGL3 1415-1815 R | CCCAAGCTTGGTGCTGGTGCGCAGAC |  |
| pGL3 1600-1815 F | CGAGCTCGCCACCTGCACGTCGCATGG | Cloning PCR |
| pGL3 1600-1815 R | CCCAAGCTTGGTGCTGGTGCGCAGAC |  |
| pGL3 1660-1815 F | CGAGCTCGCCACCGAGGACTCTTGGAC | Cloning PCR |
| pGL3 1660-1815 R | CCCAAGCTTGGTGCTGGTGCGCAGAC |  |
| pGL3 1715-1815 F | CGAGCTCGCCACCGACTGTTTGTTTAA | Cloning PCR |
| pGL3 1715-1815 R | CCCAAGCTTGGTGCTGGTGCGCAGAC |  |
| pGL3 1415-1715 F | CGAGCTCGCCACCGACGTCCTTTGTTT | Cloning PCR |
| pGL3 1415-1715 R | CCCAAGCTTCTTTGAAGTATGCCTCA |  |
| HNF1α-F | TCTACAGCCACAAGCCCGAG | qRTPCR |
| HNF1α-R | GAGGTGAAGACCTGCTTGGT |  |
| HNF4α-F | GGCCAAGTACATCCCAGCTT | qRTPCR |
| HNF4α R | TCATTGCCTAGGAGCAGCAC |  |
| PGC1α-F | TCCTCACAGAGACACTAGACAG | qRTPCR |
| PGC1α-R | CTGGTGCCAGTAAGAGCTTCT |  |
| C/EBPα-F | TGGACAAGAACAGCAACGAGTA | qRTPCR |
| C/EBPα-R | ATTGTCACTGGTCAGCTCCAG |  |
| hGAPDH-F | CCACCCATGGCAAATTCCATGGCA | qRTPCR |
| hGAPDH-R | TCTAGACGGCAGGTCAGGTCCACC |  |
| 3.5 kb RNA-F | GCCTTAGAGTCTCCTGAGCA | qRTPCR |
| 3.5 kb RNA-R | GAGGGAGTTCTTCTTCTAGG |  |
| EnhII/CP-F | ACTGTTTGTTTAAAGACTGGGAG | CHIP PCR |
| EnhII/CP-R | GGTGCTGGTGCGCAGACCAATTTA |  |
| Mice GAPDH-F | ATGGTGAAGGTCGGTGTGAA | qRTPCR |
| Mice GAPDH-R | CGCTCCTGGAAGATGGTGAT |  |
| IFI6Q-F | AAGGCGGTATCGCTTTTCTT | qRTPCR |
| IFI6Q-R | ATCGCAGACCAGCTCATCA |  |

**Table 2: The sequence of probes used in this study**

| **Name** | **Sequence (5’-3’)** | **use** |
| --- | --- | --- |
| EnhII1715-1770 | ACTGTTTGTTTAAAGACTGGGAGGAGTTGGGGGAGGAGATTAGGTTAAAGGTCTT AGTGAAAGTGGAGAAGTGAAAGTG | EMSA |
| EnhII 1415-1470 | GACGTCCTTTGTTTACGTCCCGTCGGCGCTGAATCCTGCGGACGACCCTTCTCGG | EMSA |
| EnhII 1760-1815 | GACGTCCTTTGTTTACGTCCCGTCGGCGCTGAATCCTGCGGACGACCCTTCTCGG | EMSA |
